# Some Vibrational-Rotational Bands of Deuterated Methanes^1^

**DOI:** 10.6028/jres.063A.007

**Published:** 1959-10-01

**Authors:** Harry C. Allen, Earle K. Plyler

## Abstract

A parallel band at 2,200 cm^−1^ and a perpendicular band at 2,780 cm^−1^ of CH_3_D have been observed under high resolution and analysed. The analysis of the perpendicular band revealed the presence of l-type doubling in the doubly degenerate excited state. From the analysis of the parallel band it is found that *B*_0_= 3.880 cm^−1^. A hybrid band of CD_3_H has been observed near 2,600 cm^−1^. Both active components, *A* and *E* are observed and analysed. The ground state *B*_0_ value found from this analysis is in good agreement with previous determinations.

## 1. Introduction

A study of the spectra of deuterated methanes was undertaken as part of a program for studying the spectra of deuterated molecules. In spite of the apparent simplicity of the CH_3_D and CD_3_H molecules, both being symmetric tops, very little work has been done on their spectra. Early work on the spectra of these molecules was done at rather low resolution [1,2].^2^ More recently one band of each molecule has been studied under somewhat higher resolution by Thompson and his coworkers [3,4]. Two bands of CD_3_H have been studied in the photographic infrared [5] and the overtone of the C—H stretch, *v*_1_ has been studied with high resolution [6]. A complete study of the spectra of the two molecules has been made using prism instruments by Wilmshurst and Bernstein [7].

The resolution available with the instruments of the Radiometry Section made it feasible to study several bands of these molecules between 3 and 6 *μ* in greater detail than had been possible before. No perpendicular band of either molecule had previously been studied with high resolution. In this work a perpendicular band of CH_3_D was observed and analysed. Likewise a hybrid band of CD_3_H was observed and analysed, one component of which obeys the selection rules of a perpendicular type band.

## 2. Experimental Method

The spectra were recorded on a 2.35-m grating spectrometer. For the wavelengths shorter than 4 *μ* the spectrometer was equipped with a 10,000 lines/in. grating which was double-passed, and a cooled PbS detector. Higher orders of the grating were eliminated by using a cut-off filter that started transmitting at 2.7 *μ.* The resolution in this region is 0.02 to 0.03 cm^−1^. For the CH_3_D band at 2,200 cm^−1^ the spectrometer was equipped with a PbTe detector and the grating was not double-passed. The resolution in this band was ~0.05 cm^−1^. The spectra were measured by using the fringes of a Fabry-Perot interferometer as previously described [8].

The CH_3_D and CD_3_H were obtained from Merck and Co., Ltd., and had a stated purity of 98 percent. The gases were used without further purification and no troublesome impurities were found. The gas was placed in a cell with a pathlength of 6 m and the gas pressures used ranged from a few millimeters to a few centimeters of Hg.

**Figure 1:**
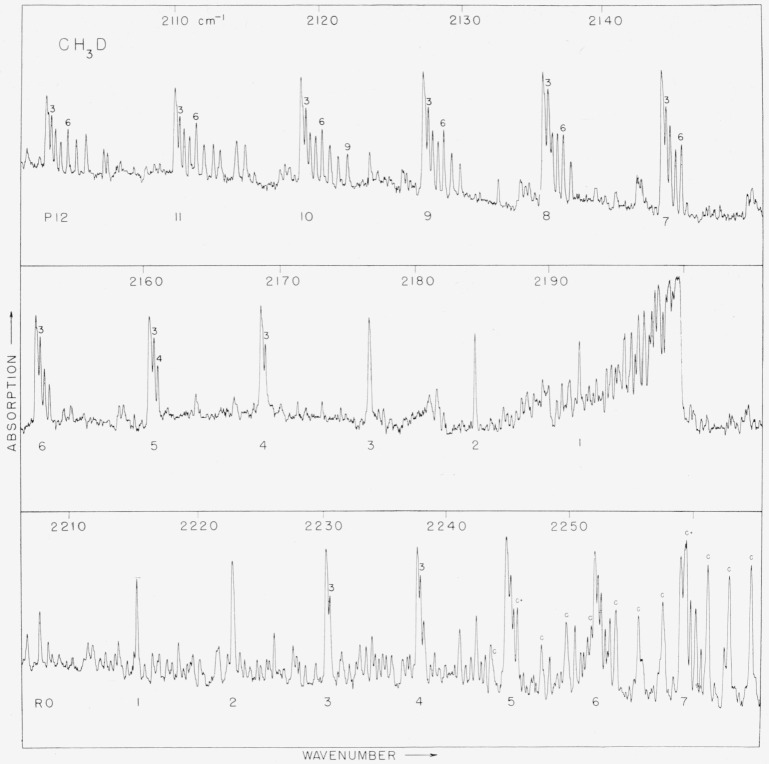
The parallel band, v_2_, of *CH_3_D* at 2,200 cm^−*1*^.

**Figure 2:**
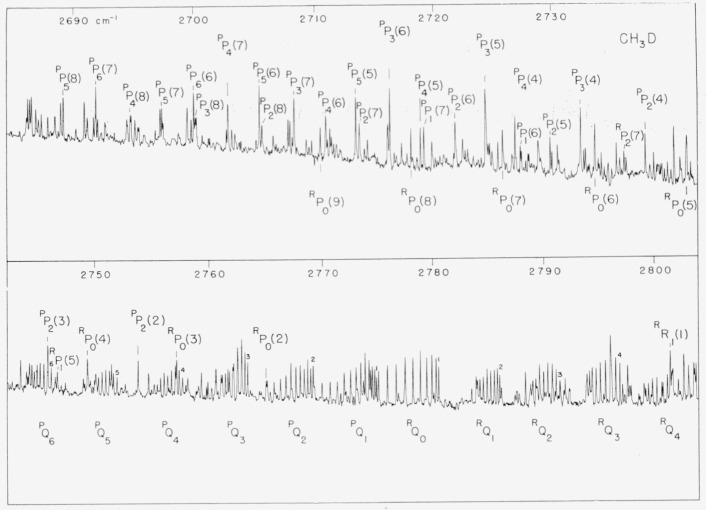
The perpendicular band, v_3_+v_5_ of *CH_3_D*. The transitions have been designated by the notation suggested by Herzberg. The presuperscript *P* or *R* means Δ*K*=−1 or Δ*K*=+1 respectively. The basic symbol *P*, *Q*, or *R* has the usual meaning Δ*J*=−1, 0, +1. The su script gives the *K* value of the ground state level of the subband while the number in parenthesis gives the *J* value of the ground state.

Two regions of absorption of CH_3_D were studied in detail. The parallel band at 2,200 cm^−1^ which is, in Herzberg’s notation [9], *v*_2_, and a perpendicular combination band near 2,780 cm^−1^ which has the excited state *v*_3_+*v*_5_. Recorder traces of the observed spectra of CH_3_D are shown in figures 1 and 2.

**Figure 3:**
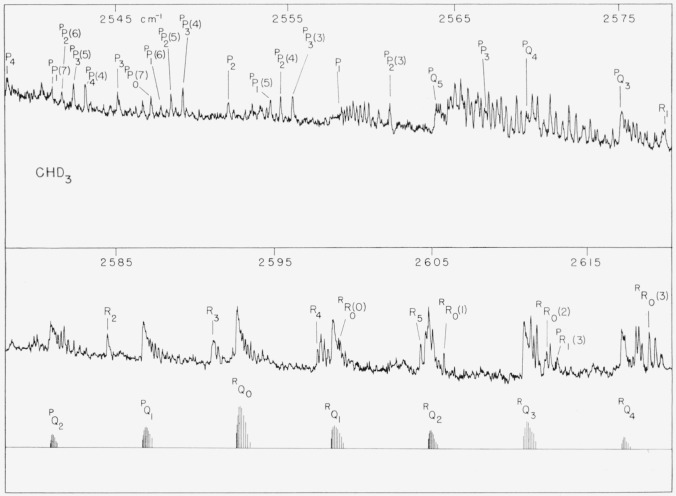
A portion of the *2*v_5_ band of *CD_3_H* showing the Q-branch and low J-, P-, and R-branch transitions of the A-component. The *Q*-branches of the *E*-component are also clearly evident in the lower panel.

**Figure 4:**
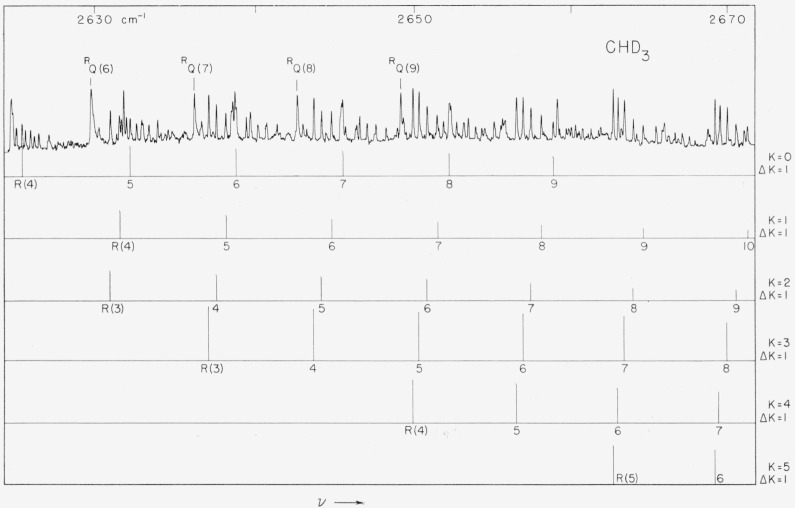
The *^R^R_K_* branches of the *E*-component of the *2*v_5_ band of *CD_3_H*.

One region of CD_3_H was studied in detail. This region is of particular interest since the absorption arises from a hybrid band. Both infrared active components of this band arc resolved. The observed absorption is shown in figures 3 and 4.

## 3. CH_3_D

### Parallel band

The absorption in the 2,200 cm^−1^ region is clearly that arising from a typical parallel band. Unfortunately the short wavelength portion of the absorption is masked by the absorption of atmospheric CO_2_. In spite of this handicap it is possible to identify transitions as high as *R*(10), although some of the *K*-components are not observed because of the atmospheric absorption. The observed wave numbers and quantum number assignments are given in table 1. Some of the assignments are indicated on figure 1. The intensity alternation of the *K*-components arising from the nuclear spin statistics of the three identical H-atoms is clearly seen in figure 1. The statistical weights of the *A* and *E* rotational levels are in the ratio 2:1, and the observed intensity alternation simplifies the process of assigning quantum numbers. The rotational- vibrational energy levels of a symmetric rotor in a nondegenerate vibrational state are given by

$$\begin{array}{l} F(J,K)=BJ(J+1)+(A-B)K^{2}\\ \;+D_{J}J^{2}{\left(J+1\right)}^{2}+D_{JK}J(J+1)K^{2}+D_{K}K^{4}. \end{array}$$ (1)

This energy expression together with the selection rules

$$\begin{array}{lll} \Delta J=0,\pm 1 & \Delta K=0 & K\neq 0\\ \Delta J=\pm 1 & \Delta K=0 & K=0 \end{array}$$

enables the calculation of the difference relations involving only the ground state energies.

$$\begin{array}{l} \Delta F_{2}^{\prime \prime }=\frac{R(J-1,K)-P(J+1,K)}{\left(2J+1\right)}=(2B^{\prime \prime }-3D_{J}^{\prime \prime })\\ \;-4D_{J}^{\prime \prime }{\left(J+\frac{1}{2}\right)}^{2}-4D_{JK}^{\prime \prime }K^{2}, \end{array}$$ (2)

and a similar one involving only excited state energies,

$$\begin{array}{l} \Delta F\prime_{2}=\frac{R(J,K)-P(J,K)}{\left(2J+1\right)}=(2B\prime -3D\prime_{J})\\ \;-4D\prime_{J}{\left(J+\frac{1}{2}\right)}^{2}-2D\prime_{JK}K^{2}, \end{array}$$ (3)

which were used to determine the B′s and centrifugal distortion constants. Since the transitions in the P- and R-branches with *K*= 0, 1, 2 were not resolved these transitions were not used in the calculations. Thus 35 equations of type (2) and 29 equations of type (3) with *K*≥3 were solved by the method of least squares for the best estimates of the constants. The combination sum,

$$R(J-1,K)+P(J,K)=2v_{0}+2[(A\prime -B\prime )-(A^{\prime \prime }-B^{\prime \prime })]K^{2}+2(D_{K}^{\prime }-D_{K}^{\prime \prime })K^{4}+2[(B\prime -B^{\prime \prime })+(D_{JK}^{\prime }-D_{JK}^{\prime \prime })K^{2}]J^{2}+2(D\prime_{J}-D_{J}^{\prime \prime })J^{2}{\left(J+1\right)}^{2},$$ (4)

was used to determine the band center. The results of these determinations are given in table 2. These molecular constants compare favorably with previous results [4] although a different method of analysis was used here. The present results are believed to be more precise and are more complete than those reported previously for this band. Earlier workers neglected the effect of *D_JK_* which accounts for the apparently different values of (*A*′–*B*′)–(*A*″–*B*″) in the *P*- and *R*-branches, a result they noticed but did not explain. Starting at P(10) there is a perturbation which affects the *K*=9 component. This arises from an interaction of the excited state with another higher vibrational level of CH_3_D. Some of the transitions to this other level can be seen in figure 1. Unfortunately not enough of this overlapping band can be identified to enable a complete understanding of this perturbation. However such a perturbation cannot have any effect on the ground state constants as determined by the method outlined here. The perturbed levels were not used in determining the excited state constants.

### Perpendicular band

The absorption near 2,780 cm^−1^ arises from the perpendicular band *v*_3_+*v*_5_, and is shown in figure 2. The excited state of this band is doubly degenerate and the rotational contribution is not given by the simple expression (1). The levels are perturbed by the interaction of rotation and vibration which leads to an additional term in the energy, thus,

$$F(J,K)=BJ(J+1)+(A-B)K^{2}\mp 2A\zeta K+\text{centrifugal terms}.$$ (5)

For a perpendicular transition the selection rules on *K* are somewhat relaxed,

$$\begin{array}{cc} \Delta J=0,+1 & \Delta K=\pm 1. \end{array}$$

The overall band is thus made up by a series of subbands. [9, pp. 425 and 431] The prominent feature of the overall band is the series of *Q*-branches of the subbands whose spacing is approximately 2[*A*′(1–*ζ*)–*B*′

The series of *Q*-branches characteristic of a perpendicular band are clearly evident in the 2,780 cm^−1^ region. Again the nuclear spin statistics provide the clue which enables the assignment of *K*-values to the *Q*-branches. Those *Q*-branches with ground state *K*’s a multiple of three are stronger due to the nuclear spin statistics and this intensity pattern is clearly evident in the figure. This is the first perpendicular band to be observed in which all the individual *Q*-branch transitions are resolved. Once the quantum numbers have been assigned in the *Q*-branches, each subband can be considered separately and effective constants can be calculated from the *Q*-branch using formulas applicable to linear molecules, i.e.,

$$v=v_{\text{sub}}+(B\prime -B^{\prime \prime })J(J+1)+(D\prime_{J}-D_{J}^{\prime \prime })J^{2}{\left(J+1\right)}^{2}.$$ (6)

These effective constants together with the ground state constants determined from the parallel band can then be used to calculate the *P*- and *R*-branches of the subband associated with the particular *Q*-branch in question by using

$$v=v_{\text{sub}}+(B\prime +B^{\prime \prime })m+(B\prime -B^{\prime \prime })m^{2}+2(D\prime_{J}+D_{J}^{\prime \prime })m^{3}+(D\prime_{J}-D_{J}^{\prime \prime })m^{4},$$ (7)

with *m=−J* for the *P*-branch and *m=J+*1 for the *R*-branch.

It should be remembered that in a given subband the branch in which Δ*J*=Δ*K* is the stronger and becomes very much stronger than the branch with Δ*J*≠Δ*K* as *K* increases. In this manner assignments could be made for all the subbands except that for which *K*=0. The observed wave numbers and quantum number assignments made in this manner are given in table 3. Unfortunately the short wavelength end of the band is overlapped by a much stronger band of CH_3_D so the weaker perpendicular band could not be analyzed in this region. The *P*- and *R*-branch transitions of the *K*=0 subband were located by a trial and error procedure. Since both branches are fairly strong in this molecule they could be picked out with a minimum of difficulty.

Ground state constants and band centers were determined using equations (2) and (4) with *K*=0. The *v*_0_ found from (4) agreed exactly with that found from (6) but the values of *B*′–*B*″ were different by nearly a factor of 3, that found using (6) being larger. This behavior is reminiscent of the behavior of a perpendicular transition of a linear molecule where the levels of the degenerate state have the so-called l-type doubling, one component of the doublet being accessible from the ground state with Δ*J*=0, and the other component being accessible from the ground state when Δ*J*=±1, thus leading to a different effective *B*′ value for the *Q*-branch than for the *R*- and *P*-branches.

The possibility of this type of behavior in symmetric top molecules was first suggested by Wilson [9, 10]. The interaction to a second order approximation has been worked out by Nielsen [11] who has found that for molecules with *C*_3_*_v_* symmetry the interaction matrix elements are of the form

$$(v_{i},l_{i},K|H_{2}^{\prime }|v_{i},l_{i}\pm 2,K\pm 2=q{\left\{\left[(v_{i}\mp l_{i})(v_{i}\pm l_{i}+2)]\times [J(J+1)-K(K\pm 1)][J(J+1)-K(K\pm 1)(K\pm 2)\right]\right\}}^{\frac{1}{2}}$$ (7)

As can be seen levels are mixed pair-wise by this interaction but only the levels *K*=1, *l*=1; *K*=−1, *l*=−1 are actually split. All other levels are merely shifted in position by this interaction to this approximation. For *K*=1, *l*=1, (7) reduces to

q[v+1]J(J+1) (8)

which is the same as the expression for a linear molecule. The actual splitting is twice the quantity given in (8). Since the interaction in the *K*=0 subband varies as *J*(*J*+1) it has the effect of producing two effective *B*′ values in the subband. Thus from the two effective *B*′s found for the excited state with *K*=1 it is possible to evaluate *B′–B*″ and *q* for this state. These results are included in table 5.

Sufficient lines in the *P*- and *R*-branches of 6 subbands were observed to enable a determination of subband centers from the transitions using the relation

$${\;}^{R}R_{K}(J-1){+}^{R}P_{K}(J)=2v_{\text{sub}}+(B\prime -B^{\prime \prime })J^{2}+(D\prime -D^{\prime \prime })J^{4}$$ (9)

since *K* is a constant for each subband. The highest value of *K* for any subband is 3. Similarly the band centers of the same subbands can be determined from (6). The *v*_0_’s obtained by these two methods agree within the experimental error and are compared in table 4.These subband centers may then be used to determine the quantities in the relation

$$\begin{array}{l} v_{\text{sub}}=v_{0}+[A\prime (1-2\zeta )-B\prime ]\pm 2\\ {}[A\prime (1-\zeta )-B\prime ]K+[(A\prime -B\prime )-(A^{\prime \prime }-B^{\prime \prime })]K^{2} \end{array}$$ (10)

This relation neglects the effect of centrifugal distortion but this should not cause serious error since only values of *K*≤3 are used. The main error will arise because nowhere is $D_{jK}^{\prime }$ considered. However since no high quantum numbers could be used in the analysis there was no good way to determine this constant with any precision for the excited state.

In view of the perturbation in the excited state, the usual combination relations among the transitions of a perpendicular band could not be used in the analysis of this band [12]. However, if the perturbation discussed above is the only perturbation present, the combination relation

$$\begin{array}{l} {[}^{P}Q(J_{K}){+}^{R}Q(J_{K})]/2=v_{0}+[A\prime (1-2\zeta )-B\prime ]\\ \;+(B\prime -B^{\prime \prime })J(J+1)+(D\prime -D^{\prime \prime })J^{2}{\left(J+1\right)}^{2}, \end{array}$$ (11)

*K* constant, averages out this perturbation and if for each *K* one plots

$${[}^{P}Q(J_{K}){+}^{R}Q(J_{K})]/2-(D\prime -D^{\prime \prime })J^{2}{\left(J+1\right)}^{2}\;\text{versus}\;J(J+1)$$

a straight line should result. Such a plot for three values of *K* gives good straight lines and hence seems to rule out any further perturbations.

The value of *q* obtained from the *K*=0 subband does not appear to be entirely satisfactory for the other subbands. However, not enough transitions in the other subbands are observed to enable one to make meaningful higher order corrections. The constants derived from the analysis of this band are collected in table 5. Unfortunately the inadequacy of the data makes these constants subject to uncertainties which are larger than would be normal for measurements of this precision. The strong overlapping in the high frequency portion of the absorption sharply limits the number of *^R^R* and *^P^R* lines which can be assigned and the general weakness of the *^R^P* and *^P^R* lines makes it impossible to assign many of these transitions. However it is felt that the analysis is correct in all essential features and that only the evaluation of the small higher order corrections needs improvement.

If A″ is calculated from the methane geometry, it is then possible to evaluate *ζ* and *v*_0_. Using *A*=5.24 cm^−1^ one finds *ζ*=−0.26. Simple theory predicts [13] that the *ζ* of this band should be the same as for the fundamental *v*_5_. Herzberg [9] has estimated *ζ*_5_= −0.27, in remarkable agreement. However, it is felt that this may be in large measure fortuitous. The above figures lead to a value of *v*_0_=2,776.33 cm^−1^.

## 4. CD_3_H

### Hybrid band

Only the absorption of CD_3_H near 2,600 cm^−1^ was studied in detail. This region has more than usual interest. The main absorption here is due to the first overtone of *v*_5_. *v*_5_ is a degenerate fundamental and its first overtone has the components *A*_1_ and *E* [9,14].

The electric dipole moment selection rules allow transitions from the ground state to both the *A*_1_ and *E* components of this overtone level. Thus one would expect the absorption in this region to show the features of both a parallel and a perpendicular case. This is, indeed, the case as can be seen in figures 3 and 4. There is a collected *Q*-branch near 2,565 cm^−1^ which is characteristic of a parallel type band. It is possible to identify the *P*- and *R*-branch transitions of this band and they are labelled in figures 3 and 4. Unfortunately the absorption is very weak so the *K*-components of the *P*- and *R*-branch transitions could not be identified but only the general absorption peak representing the aggregate absorption of all the *K*-components. Thus precise molecular constants could not be determined for this component of the overtone. The band center of this component is 2,564.6 cm^−1^. The *B*_0_ value is compatible with the previously determined values, 3.278 cm^−1^ and the *B′–B*″ value is about 0.01 cm^−1^.

The series of *Q*-branches of the subbands belonging to the *E*-component are clearly evident in figure 3, the strongest being near 2,590 cm^−1^. This strongest *Q*-branch belongs to the *K*=0 subband, and the other *K*-numbering follows automatically, since again for this molecule the subbands with *K* a multiple of 3 are strong due to nuclear spin statistics. In this case the ratio is 11:8. Unfortunately the structure of these *Q*-branches cannot be resolved well because the band is very weak, and if the pressure in the absorption cell is increased the pressure broadening eliminates the fine structure. Also the quantity (*B′–B″*) is very small in this transition ~0.007 cm^−1^.

The assignments of the *P*- and *R*-branch transitions were made by calculating the approximate line positions using the relation

$$\begin{array}{c} v_{0}=v_{sub}\pm [A\prime (1-\zeta )-B\prime ]K+[(A\prime +B\prime )-(A^{\prime \prime }-B^{\prime \prime })]\\ K^{2}\pm (B\prime +B^{\prime \prime })m+(B\prime -B^{\prime \prime })m^{2} \end{array}$$ (12)

Here the negative sign of the *K* dependent term applies for Δ*K*=−1 and *m=−J* for the *P*-branch and *m*=*J+*1 for the *R*-branch. The coefficients of *K* and *K*^2^ were estimated from the *Q*-branch positions and the coefficients of *m* and *m^2^* were estimated from previous work on this molecule [5, 6]. The resulting assignments are given in table 6 and shown in figures 3 and 4. Assignments could be made in 11 subbands. Unfortunately, due to weakness of the absorption only lines in the branches Δ*K*=Δ*J* could be assigned with any degree of assurance and are the only ones given in the table and the only ones used in subsequent calculations. In the *K*=0 subband most of the *P*-branch lines were masked by other absorption and could not be uniquely assigned to absorption peaks. The measurements could not be extended to longer wavelengths because of the weakness of the absorption and the fact that the sensitivity of the PbS detector falls off rapidly beyond 3.5 *μ*. Since the *Q*-branch transitions were not resolved and since, in general, only the stronger branch of the subbands could be observed it was not possible to use the usual combination relations for a perpendicular band in the analysis. Thus an alternate and less desirable method of deriving the molecular constants had to be used. This method involved the use of an expression for the line position deduced from the energy expression for the two energy levels in the transition. In order to include the effects of centrifugal distortion an expression of thirteen terms had to be used. One would expect to fit almost any set of observations with this many disposable constants and the use of this expression is justified only by the results obtained. The expression used is

$$\begin{array}{l} v=v_{0}+[A\prime (1-2\zeta )-B\prime ]+bK+cK^{2}+dK^{3}+eK^{4}\\ +fm+gm^{2}+hm^{3}+im^{4}+jmK+kmK^{2}+1m^{2}K+nm^{2}K^{2} \end{array}$$ (13)

in which the coefficients are readily related to the molecular constants from the expressions for the vibrational rotational terms, (1) and (5). Eighty-one equations of type (13) were fit by the method of least squares. The resulting values of the molecular constants, where they can be compared, are in remarkable agreement with those determined previously [5, 6]. The agreement between observed and calculated frequencies about 0.01 cm^−1^, and the uncertainties in the constants are very small. The derived constants are given in table 7. The size of the higher order terms indicates that there is no l-type doubling interaction in the excited state of this band. Using the methane geometry it is possible to calculate a value for the ground state *A* of this molecule, *A*=2.628 cm^−1^. With this value of *A*″ and the constants in table 7 one finds a value *ζ*=0.89. Thompson [4] has estimated *ζ*_5_ from the fundamental to be 0.67. Simple theory predicts the *ζ* here should be twice the *ζ* of the fundamental; however, it is thought that the *ζ* value from the fundamental band is not sufficiently precise to justify comparisons at this time.

The band center, *v*_0_, becomes 2,592.64 if one uses the above values of *A*′ and *ζ*. The separation of these two components is given by *g*_55_[(l+2)^2^−l^2^], with l=0 in this case. Thus from the component separation *g*_55_=8.05 cm^−1^.

## Figures and Tables

**Table 1:** Observed wave numbers of 2,200 cm^−*1*^ band of *CH_3_D*

*J_K_*	*R*	*P*
		
0_0_	2,207. 70	………
1_0_	………	2,192.29
1_1_	2,215. 32	………
2_0,1_	2,222. 82	2,184.45
2_2_	2,222. 93	………
3_0,1_	2,230. 23	2,176.53
3_2_	2,230. 35	2,176.63
3_3_	2,230. 54	………
4_0,1_	2,237. 56	2,168. 50
4_2_	2,237. 66	2,168.63
4_3_	2,237. 86	2,168.84
4_4_	2,238.13	………
5_0,1_	2,244. 78	2, 160.44
5_2_	2,244.89	2,160. 55
5_3_	2,245.09	2,160. 77
5_4_	2, 245. 34	2,161.04
5_5_	2,245. 68	………
6_0,1_	2,251. 94	2,152.26
6_2_	………	2,152. 39
6_3_	2,252. 23	2, 152.59
6_4_	2,252. 48	2,152.87
6_5_	2, 252. 80	2,153.23
6_6_	2, 253. 21	………
7_0,1_	2, 258.97	2,144.02
7_2_	2,259.05	2,144.13
7_3_	2, 259. 31	2,144.35
7_4_	………	2,144.63
7_5_	2, 259. 84	2,144.98
7_6_	2,260. 25	2,145.42
7_7_	2,260. 71	………
8_0,1_	2, 260. 90	2,135. 68
8_2_	2, 266. 02	2,135.81
8_3_	2,266. 20	2,136.03
8_4_	2, 266. 47	2,136.31
8_5_	………	2,136.37
8_6_	2,267.18	2,137.10
8_7_	2,267.66	2,137. 61
9_0,1_	2,272. 75	2,127.29
9_2_	2,272.85	2,127. 41
9_3_	2,273.05	2,127.63
9_4_	2,273.31	2,127.92
9_5_	………	2,128.28
9_6_	2,274.00	2,128. 72
9_7_	2,274.47	2,129.25
9_8_	2,274.99	2,129.83
10_0.1_	2,279.48	2,118. 83
10_2_	2,279. 58	2,118. 95
10_3_	2,279.80	2,119.16
10_4_	2,280.04	2,119. 44
10_5_	………	2,119. 80
10_6_	2,280. 75	2,120.26
10_7_	2,281.21	2,120. 78
10_8_	………	2,121.38
10_9_	………	2,121.99
11_0,1_	………	2,110.27
11_2_	………	2,110. 40
11_3_	………	2,110. 60
11_4_	………	2,110.90
11_5_	………	2,111.26
11_6_	………	2, 111. 70
11_7_	………	2,112. 23
11_8_	………	2,112.84
11_9_	………	2,113.30
11_10_	………	2,114.33
12_0,1_	………	2,101.62
12_2_	………	2,101. 77
12_3_	………	2,101.97
12_4_	………	2,102.27
12_5_	………	2,102. 65
12_6_	………	2,103.10
12_7_	………	2,103. 62
12_8_	………	2,104. 25
12_9_	………	2,105.48
12_10_	………	2,105. 75

**Table 2:** Constants of *CH*_3_*D* from v_2_ at 2,200 cm^−*1*^

	Excited	Ground
		
B′	3.837_8_ cm^−1^	3. 880_0_ cm^−1^
$C_{J}^{\prime }$	0.00005_5_	0.00005
$D_{\mathrm{JK}}^{\prime }$	.00012	.00012

(*A′–B*′) – (*A″–B″*)=0.03858 *v*_0_=2200.03.

**Table 3:** Subbands of *CH_3_D* perpendicular band at 2,780 cm^−*1*^

J	K=0	K=1		K=2		K=3		K=4	K=5	K=6
									
	ΔK=1	ΔK=1	ΔK=−1	ΔK=1	ΔK=−1	ΔK=1	ΔK =−1	ΔK=−1	ΔK=−1	ΔK =−1
										
Δ*J*=0:										
1	2,780.42	………	2,774.85	………	………	………	………	………	………	………
2	2,780.20	2,785.96	2,774.70	………	2,769.15	………	………	………	………	………
3	2,779.82	2,785.74	2,774.48	2,791.43	2,768.91	………	2,763.40	………	………	………
4	2,779.35	2,785.44	2,774.19	2,791.11	2,768.64	2,796.86	2,763.14	2,757.66	………	………
5	2,778.78	2,785.11	2,773.85	2,790.75	2,768.33	2,796.44	2,762.82	2,757.38	2,751.91	2,746.15
6	2,778.13	2,784.78	2,773.45	2,790.34	2,767.98	………	2,762.47	2,757.04	2,751.59	2,745.85
7	2,777.40	2,784.43	2,773.00	2,789.95	2,767.60	2,795.54	2,762.09	2,756.71	2,751.27	2,745.50
8	2,776.61	………	2,772.52	2,789.57	2,767.18	2,795.11	2,761.64	2,756.37	2,750.92	2,745.19
9	2,775.82	………	2,771.95	2,789.23	2,766.73	2,794.70	2,761.14	2,756.05	2,750.61	2,744.90
10	2,775.04	………	2,771.32	………	2,766.23	………	2,760.58	………	2,750.30	………
Δ*J=+*1:										
0	2,788.27	………	………	………	………	………	………	………	………	………
1	2,795.99	2,801.48	………	………	………	………	………	………	………	………
2	2,803.64	2,809.00	2,797.76	2,814.70	2,792.19	………	………	………	………	………
3	2,811.28	2,816.47	2,805.26	2,822.15	2,799.68	2,827.87	2,794.16	………	………	………
4	2,818.93	2,823.89	2,812.65	2,829.51	2,807.12	2,835.20	2,801.62	………	………	………
5	2,826.61	2,831.35	2,820.00	2,836.85	2,814.50	2,842.51	2,809.00	………	………	………
6	2,834.32	2,838.71	2,827.29	2,844.20	2,821.83	………	2,816.29	………	………	………
Δ*J*=−1:										
1	………	………	………	………	………	………	………	………	………	………
2	2,764.99	………	2,759.33	………	2,753.76	………	………	………	………	………
3	2,757.15	………	2,751.44	………	2,745.85	………	2,740.35	………	………	………
4	2,749.35	2,754.70	2,743.47	………	2,737.91	………	2,732.39	2,726.91	………	………
5	2,741.47	2,746.67	2,735.43	2,752.36	2,729.89	2,758.13	2,724.40	2,718.91	2,713.46	………
6	2,733.63	2,738.61	2,727.34	2,744.27	2,721.83	2,749.96	2,716.34	2,710.89	2,705.43	2,700.03
7	2,725.85	2,730.54	2,719.20	2,736.12	2,713.76	2,741.78	2,708.27	2,702.85	2,697.39	2,691.97
8	2,718.12	………	2,711.05	2,727.97	2,705.64	2,733.63	2,700.14	2,694.77	2,689.32	2,683.91
9	………	………	………	………	………	………	………	………	2,681.27	………
10	………	………	………	………	………	………	………	………	2,673.17	………

**Table 4:** Subband centers of v_*3*_+v_*5*_ band of *CH_3_D*

*K*	Δ*K*	*Q*-branch	*R*-and *P*-branches
			
0	1	2,780.55	2,780.55
1	1	2,786.15	2,786.17
1	−1	2,774.90	2,774.88
2	1	2,791.90	2,791.90
2	−1	2,769.33	2,769.31
3	1	2,797.73	………
3	−1	2,763.76	2,763.75
4	−1	2,758.30	………
5	−1	2,752.77	………

**Table 5:** Derived constants from the v_*3*_ + v_*5*_ band of *CH_3_D*

		*cm* ^−1^
*v*_0_+[*A*′(1−2*ζ*)−*B*′]	=	2,780.55
*A*′(1−*ζ*)−*B*′	=	2.82
(*A*′−*B*′)−(*A*″−*B*″)	=	0.02
*B*″	=	3.88
*B*′−*B*″	=	−0.041
$D\prime_{J}-D_{J}^{\prime \prime }$	=	.00011
*q*	=	.011

**Table 6:** Subband assignments for the *E*-component of the hybrid band of *CD_3_H*

*J*	*K*=0	*K*=1		*K*=2		*K*=3		*K*=4		*K*=5		*K*=6
										
		Δ*K*=+1	Δ*K*=−1	Δ*K*=1	Δ*K*=−1	Δ*K*=1	Δ*K*=−1	Δ*K*=1	Δ*K*=−1	Δ*K*=1	Δ*K*=−1	Δ*K*=1
												
Δ*J*=+1:												
0	2,599.19	………	………	………	………	………	………	………	………	………	………	………
1	2,605.76	………	………	………	………	………	………	………	………	………	………	………
2	2,612.36	………	………	………	………	………	………	………	………	………	………	………
3	2,618.98	………	2,613.06	*2*,624.40	………	………	………	………	………	………	………	………
4	2,625.58	………	2,619.70	2,630.39	………	2,637.12	………	………	………	………	………	………
5	2,632 20	2,631.57	2,626.33	2,637.59	………	2,643.70	………	2,649,89	………	………	………	………
6	2,638.84	2,638.16	………	2,644.19	………	2,650.29	………	2,656.48	………	2,662.74	………	………
7	2,645.52	2,644.79	………	2,650.81	………	2,656.89	………	2,663.06	………	2,669.31	………	2,675.64
8	2,652.17	2,651.43	………	2,657.41	………	2,663.48	………	2,669.64	………	2,675.86	………	2,682.18
9	………	2,658.07	………	2,664.03	………	2,670.08	………	2,676.21	………	2,682.42	………	2,688.71
10	………	2,664.72	………	2,670.66	………	2,676.69	………	2,682.80	………	2,688.99	………	2,695.27
11	………	2,671.38	………	2,677.31	………	2,683.32	………	2,689.39	………	2,695.55	………	2,701.80
12	………	2,678.07	………	2,683.98	………	2,689.93	………	2,695.97	………	2,702.12	………	2,708.36
13	………	………	………	………	………	2,696.55	………	2,702.58	………	2,708.67	………	2,714.90
14	………	………	………	………	………	2,703.19	………	2,709.18	………	………	………	2,721.42
15	………	………	………	………	………	2,709.83	………	………	………	………	………	2,727.99
Δ*J*=−1:												
1	………	………	………	………	………	………	………	………	………	………	………	………
2	………	………	………	………	………	………	………	………	………	………	………	………
3	2,572.99	………	2,567.08	………	2,561.21	………	2,555.42	………	………	………	………	………
4	………	2,572.45	2,560.58	………	2,554.71	………	2,548.94	………	2,543.21	………	………	………
5	2,559.98	2,565.96	2,554.09	………	2,548.24	………	2,542.48	………	2,536.74	………	2,531.09	………
6	………	2,559.50	2,547.62	………	2,541.80	………	2,536.06	………	2,530.28	………	2,524.63	………
7	………	………	2,541.21	………	2,535 39	………	………	………	2,523.86	………	2,518.19	………
8	………	………	2,534.83	………	………	………	………	………	………	………	2,511.78	………
9	………	………	………	………	………	………	………	………	………	………	2,505.39	………

Lines not listed are obscured by other absorption in this band.

**Table 7:** Molecular constants of *CD_3_H* derived from the *E*-component of the hybrid band near 2,600 cm^−*1*^

*A*′(1−*ζ*)−*B*′	=2.977±.002 cm^−1^
(*A*′*−B*′)−(*A″−B″*)	=0.0379±0.0018
*B*″	=3.278±0.001
*B*′	=3.285±0.001
$D_{J}^{\prime \prime }-D\prime_{J}$	=5.0±0.8×10^−5^
$D_{JK}^{\prime \prime }$	=4×10^−5^
$D_{JK}^{\prime }$	=1×10^−5^
$D_{K}^{\prime }\sim D^{\prime \prime }\sim$	~0
*v*_0_*+*[*A*′(1−2*ζ*)−*B*′]	=2592.637±0.005

## Footnotes

2 Figures in brackets indicate the literature references at the end of this paper.
